# To Label or Not to Label: California Prepares to Vote on Genetically Engineered Foods

**DOI:** 10.1289/ehp.120-a358

**Published:** 2012-08-31

**Authors:** Richard Dahl

**Affiliations:** Boston freelance writer **Richard Dahl** has contributed to *EHP* since 1995. He also writes periodically for the Massachusetts Institute of Technology.


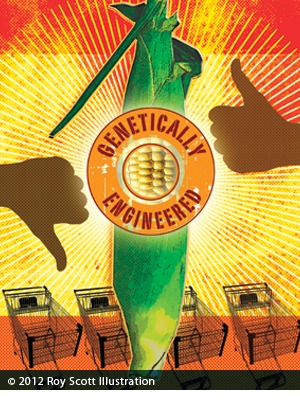
Since they were first commercially grown in the mid-1990s, genetically engineered (GE) crops have expanded across the globe, offering farmers the advantages of genetically enhanced resistance to drought, herbicides, and insects. According to the International Service for the Acquisition of Agri-Biotech Applications (ISAAA), a crop biotechnology advocacy organization, farmers in 29 countries grew nearly 400 million acres of commercial GE crops in 2011, an 8% increase from the previous year.^^1^^ An estimated 60–70% of processed foods in the United States contain GE ingredients,^^2^^ and GE corn and soybeans make up the majority of the U.S. crop.^^3^^

But while GE crop acreage has been steadily increasing, so have concerns in some quarters that producing and eating GE foods may pose unexpected environmental and health hazards. In the absence of strong health and safety data, many national governments across the world have taken steps to minimize the presence of GE food within their borders. In Europe, six nations (Austria, France, Germany, Greece, Hungary, and Luxembourg) have enacted bans on the cultivation and import of GE products,^^4^^ and nearly 50 nations worldwide require that all GE foods be labeled as such.^^5^^

In the United States, consumer concern about GE foods has been slower to surface. But that’s changing—and a ballot question this fall in California has the potential to radically alter the GE landscape throughout the rest of the United States. On 6 November 2012 California voters will decide whether foods containing genetically modified organisms (GMOs) must be labeled.

If passed, the California Right to Know Genetically Engineered Food Act—also known as Proposition 37—would require that all raw food products containing GMOs be labeled as “genetically engineered” and that any processed foods containing GMOs be labeled as “partially produced with genetic engineering” or “may be partially produced with genetic engineering,” with implementation due 1 July 2014.^^6^^ The law would exempt meat, dairy, and other products from animals that consumed feed containing GMOs but would cover such products from animals that were themselves genetically engineered. It would also exempt food sold in restaurants and alcohol.

The initiative has touched off a heated battle in the Golden State. On one side, pro-labeling advocates claim the safety of GE foods is unknown and that consumers have the right to know what’s in their food. Opposing them are an array of groups in mainstream agribusiness, the grocery industry, and the biotech industry, many of whom would bear the cost of implementing new labeling as well as potential loss of sales to wary consumers who aren’t sure what the labels mean or whether they should be worried.

Kathy Fairbanks, spokeswoman for the Coalition Against the Deceptive Food Labeling Scheme, which represents these groups, claims the initiative would “force California families to pay hundreds of dollars more in higher food prices, would cost millions in government bureaucracy, and would not provide any health and safety benefits.” The coalition also says the bill’s many exemptions make no sense and will only confuse and mislead consumers, asking on its website, “If Prop 37 was really about the ‘right to know,’ why did proponents include so many special-interest exemptions?”^^7^^

But Stacy Malkan, Fairbanks’ counterpart for the pro-labeling organization California Right to Know, says there’s no evidence to support the claim that Californians’ grocery bills would go up if the labeling measure passes. She also says that while there’s no conclusive evidence that GE foods are unsafe, there also is no conclusive evidence that they are. “Many scientists are saying that in the face of scientific uncertainty, labeling is an important tool to help track potential health risks,” she says.

Michael Hansen, a senior staff scientist at Consumers Union, offers a theoretical example of how such tracking might work: “If you take a gene from the kiwi fruit, put it into a tomato, and the tomato gets turned into sauce for your pizza, and there’s an allergic reaction, only the genetically altered tomato would produce that allergic reaction. . . . This is not like [allergy concerns associated with] conventional foods because the problem is going to be for one particular [bioengineered modification]. How are you going to figure that out unless it’s labeled? You can’t. And that’s why so many countries have labeling.”

## Accepted Technologies

Although campaigns for and against GE labeling in California are focusing heavily on economic impacts, the real debate revolves around a scientific question: Are these foods truly safe or not?

“I think it’s fair to say that most scientists think the techniques that are used [to create GE plants] are not inherently dangerous,” says Peggy Lemaux, a cooperative extension specialist in the Plant and Microbial Biology Department at the University of California, Berkeley. In fact, in 2010 the European Commission released an analysis of 50 studies conducted on GE foods over the last 25 years and concluded that GE technologies posed no greater risks than conventional breeding technologies.^^8^^

To create a GE crop strain, researchers first identify a gene from an unrelated species of plant, animal, or microbe containing the characteristics they want to transfer to the plant host. They use polymerase chain reaction to copy the gene along with promoter and terminator genes (which up- and downregulate the gene of interest) and marker genes (which signal successful genetic modification). This packet is transferred to the host plant by various means, including bacterial vectors that can penetrate cells and introduce the recombinant DNA into the host plant’s genome.^^9^^

“The reason I don’t worry about GMOs is not because someone has convinced me with a big study that they’re safe,” says Michael Eisen, an associate professor of genetics, genomics, and development at the University of California, Berkeley, and an investigator at the Howard Hughes Medical Institute. “It’s because when I look at the technology, I understand what this technology is doing. They’re introducing proteins that have been very well characterized into plants, and I don’t see any reason at all to suspect that these are harmful.” He adds, “That’s a point that people don’t seem to get: High-fructose corn syrup derived from corn that has a bacterial toxin in it to kill insects is no different from high-fructose corn syrup derived from conventional corn. It’s literally molecularly identical.”

As for actual health effects, Lemaux says of her two-part review of the literature related to GE food safety,^^10^^^^,^^^^11^^ “I haven’t found any evidence of anything on the commercial market now causing any more health problems than things that are conventionally produced or organically produced.”

A review of studies on animals fed GE plant diets also found no evidence of health hazards.^^12^^ The authors examined 24 studies, half of them multigenerational, of animals fed diets of GE maize, potatoes, soybeans, rice, or triticale (a wheat–rye hybrid). They concluded, “The studies reviewed present evidence to show that [GE] plants are nutritionally equivalent to their non-[GE] counterparts and can be safely used in food and feed.”

And in May 2012 the EU’s food safety body, the European Food Safety Authority, rejected an attempt by France to ban the planting of MON 810, an insect-resistant strain of GE maize developed by Monsanto, because there is “no specific scientific evidence, in terms of risk to human and animal health or the environment” to justify a ban.^^13^^

## Red Flags

Some scientists, however, believe questions about the safety of GE foods are far from answered. Doug Gurian-Sherman, senior scientist with the Food and Environment Program at the Union of Concerned Scientists, says the question is not whether there’s risk involved GE foods, but whether it’s greater than risks posed by conventional foods. “Because of the greater capacity to bring unknown quantities into the food supply, I’m of the school that says it has somewhat higher potential for risk,” he says. “Other scientists say no. But I don’t think it’s a settled debate.”

Hansen responds to claims that there’s no evidence of harm from GE foods by saying, “That’s just not true. I can show you all kinds of studies in the scientific literature that have . . . raised red flags that need to be followed up on.” In one such study, investigators reviewed 19 studies of mammals fed GE soybeans and maize. They found that “several convergent data appear to indicate liver and kidney problems as end points of GMO diet effects,” with the kidneys more affected in males and the liver more affected in females.^^14^^ The authors, noting the limitations of the 28- and 90-day assays they reviewed, pointed out that chronic toxicity testing is not required for GE foods, but that it should be.

Another study involved 30 pregnant and 39 nonpregnant women in Quebec and their exposure to pesticides associated with GE foods, including Cry1Ab, an insecticidal toxin produced by *Bacillus thuringiensis* (Bt) that is introduced to some GE crops to confer pest resistance.^^15^^ The researchers detected Cry1Ab in 93% of pregnant women tested, 80% of their fetuses, and 69% of nonpregnant women tested. Citing other studies that found trace amounts of Cry1Ab in the gastrointestinal contents of livestock fed GE corn, the authors suggest the toxin may not be effectively eliminated in humans—potentially a concern for the vulnerable fetus—and that eating contaminated meat may pose a risk of exposure. The human health effects of Cry1Ab exposure are unknown.

Meanwhile, Hansen also believes there’s evidence to suggest a connection between GE crops and allergenicity, which he says is good enough reason to label foods for GMOs. He points to data from the Centers for Disease Control and Prevention showing an 18% increase in reported food allergy cases among children between 1997 and 2007.^^16^^ “Is there any connection [with the concurrent increase in GE crop production]?” he asks. “You don’t know, because if there’s no labeling or any other way to tell, how would you know?”

In response to the argument that nobody’s getting sick from these crops, Gurian-Sherman says, “That’s just scientifically unsupportable. I’m not suggesting people *are* getting sick, but there’s no data to support that. Outside of acute toxicity, you can’t know that without doing epidemiology. That’s why we do epidemiology about saturated fats and trans fats and chemicals in our food—because it’s not obvious.”

## Premarket Testing

The American Medical Association (AMA) House of Delegates recently considered a proposal to endorse the labeling of GE foods but ultimately rejected it, stating that “as of June 2012, there is no scientific justification for special labeling of bioengineered foods, as a class, and that voluntary labeling is without value unless it is accompanied by focused consumer education.” The AMA instead adopted a policy statement urging “government, industry, consumer advocacy groups, and the scientific and medical communities to educate the public and improve the availability of unbiased information and research activities on bioengineered foods.”^^17^^

The AMA also took a position in favor of mandatory premarket safety testing of all GE foods. In a statement to the *Los Angeles Times* following the action, AMA Board of Trustees member Patrice Harris said, “Recognizing the public’s interest in the safety of bioengineered foods, the new policy also supports mandatory FDA [Food and Drug Administration] premarket systemic safety assessments of these foods as a preventive measure to ensure the health of the public. We also urge the FDA to remain alert to new data on the health consequences of bioengineered foods.”^^18^^ Hansen calls this support for mandatory premarket safety assessments “huge.”

Under the 1992 policy that regulates GE foods in the United States, the FDA essentially considers these foods no different than conventional foods, and premarket testing is voluntary.^^19^^ The result, critics charge, is a complete lack of transparency around the science that companies have conducted on their GE products. “There hasn’t been much independent science done or much review of the science,” Malkan says.

By the same token, says FDA spokesman Curtis Allen, “[GE foods] must meet the same legal standards, including safety standards, as foods derived from their non-GE counterparts.” Allen says firms may voluntarily indicate through labeling whether foods have or have not been developed through genetic engineering, and the policy also established a voluntary process through which producers might consult with the FDA about safety and regulatory issues before marketing the product.^^20^^

Gregory Jaffe, director of the Biotechnology Project at the Center for Science in the Public Interest in Washington, DC, says his organization has been pushing for mandatory premarket approval of GE foods for a long time, but not for labeling. “We think if there’s any question about the safety of these foods, they should not be allowed to be eaten,” he says. “They should not be allowed to be put on the market. But labeling shouldn’t be a surrogate for safety. So our view would be: If there’s any question of safety, don’t allow it in the food supply. We want to leave mandatory labeling to things that are directly related to the health and safety of existing food.”

## Weed Resistance

Although Lemaux believes GE foods themselves are safe, she does think there’s another aspect of GE production that warrants more serious attention: the environmental effects. “I think we most definitely need to be concerned about overuse of herbicides and overuse of Bt crops,” she says. “We’ve learned that before with other agricultural technologies—that if you overuse them, you get in trouble, and we’re seeing those signs now.”

Lemaux is referring to growing evidence that nature has been catching up to the ability of GE crops to defy weeds. For years, many farmers have been relying on a single tactic to manage weeds: post-emergence applications of the herbicide glyphosate to “Roundup Ready” crops that have been bioengineered to withstand the weedkiller. This approach has allowed farmers to engage in no-till agriculture, which involves minimal disturbance of the soil and thus reduces labor time as well as chemical runoff.^^21^^

But in the second decade of the GE commercialization experiment, investigators are reaching seemingly contradictory conclusions as to whether these crops actually reduce herbicide use over time.^^21^^^^,^^^^22^^ And in the last couple of years there have been numerous reports of the emergence of “superweeds” that are immune to glyphosate, forcing farmers to resort to harsher chemical measures, such as 2,4-D, an herbicide used in the defoliant Agent Orange.

Dow AgroScience has applied to the U.S. Department of Agriculture for approval to sell a new GE corn seed that is resistant to 2,4-D,^^23^^ and a decision is expected by fall 2012. The agency has received many comments criticizing Dow’s request, including one signed by a long list of farm, food, health, public interest, consumer, fishery, and environmental organizations who see only more herbicide resistance in the offing.^^24^^ “Farmers would have no interest in 2,4-D[-resistant] crops if there weren’t a raging epidemic of weeds resistant to glyphosate,” the group wrote. “Weed resistance to 2,4-D will not be prevented or even slowed by the approaches that failed so spectacularly with Roundup Ready crops: voluntary ‘stewardship’ plans and grower education.”

Meanwhile, back in California, where the GE debate is focused on the opposite end of the food-production pipeline, both sides are engaged in a pitched battle to win voters between now and Election Day. But whether the California initiative passes or not, it appears that questions about GE foods won’t be going away any day soon.

“You can never prove, in any scientifically verifiable way, that GE foods are ‘safe’—you can only verify when they are unsafe,” says Frederick L. Kirschenmann, distinguished fellow at the Leopold Center for Sustainable Agriculture at Iowa State University. “None of this means that GE foods are not safe. It just means that we do not know, because we do not know if we have thought of all the possible aspects of the innovation that might be unsafe to eat. That, from my perspective, is the strongest argument I know of for labeling—so that individuals can choose for themselves whether to eat it or not.”
